# Effectiveness of Using a Popular Media Theme in Emergency Medicine Simulation

**DOI:** 10.7759/cureus.105699

**Published:** 2026-03-23

**Authors:** Stephanie Cohen, Ross Sinicrope, Daniela Vulpe, Natalie Diers, Drake D Dixon, Elliot Richardson, Mohammed Rahman, Andrew Bobbett, Joseph Ray

**Affiliations:** 1 Emergency Medicine, University of Central Florida (UCF)/HCA Florida Osceola Hospital, Kissimmee, USA; 2 College of Medicine, University of Central Florida College of Medicine, Orlando, USA; 3 Emergency Medicine, One Brooklyn Health - Brookdale Emergency Medicine Residency, Brooklyn, USA

**Keywords:** emergency medicine medical education, graduate medical education (gme), healthcare simulation, simulation in medical education, teaching in emergency medicine

## Abstract

Introduction

Gamification is a commonly used tool in the educator’s kit to enhance engagement. Using the popular media franchise “Star Wars” as a theming and framing device to simulations may also enhance engagement, but there is potential for this same theming to promote disinterest and poor connection to the educational material if the learner is apathetic towards “Star Wars”.

Methods

Eleven residents in a community hospital residency program attended a “Star Wars” themed simulation event. A knowledge-based pre- and post-test was given along with a subjective self-analysis of the resident’s perspective on the theming and gamification of the simulation. Cohorts were divided into residents who were “Pro Star Wars” and those who were “Neutral to Star Wars” for comparison on both knowledge and self-analysis.

Results

On the subjective responses, the “Pro Star Wars” group felt that the theming made the event more engaging, enjoyable, and the learning easier than the “Neutral to Star Wars” group. Regarding gamification, a similar divide occurred with the “Pro Star Wars” group feeling more engaged, enjoying, and learning more easily through gamification. The “Pro Star Wars” group had a mean improvement on the knowledge test of 9%, and the “Neutral to Star Wars” group had a mean improvement of 7.5%, neither being statistically significant.

Conclusion

Implementation of a themed simulation, with that theme being used to frame simulations, did not have a statistically significant effect on knowledge acquisition. However, there is evidence of a subjective effect on enjoyment and engagement with learning if the learner does not resonate with the chosen theme. With mounting evidence on the positive utility of gamification and interactive learning, popular media theming is bound to be integrated into educational events. Careful design of these events needs to be done to ensure learners are not isolated and left behind.

## Introduction

Medical education based on simulation is at the heart of contemporary clinical education and provides learners with an environment to practice clinical skills and decision-making safely [1]. To improve learning, educators have begun developing gamification, which is the use of game-design features in non-game contexts [2]. Gamification has the potential to improve motivation, engagement, and enjoyment [3,4]. Thematic overlays that use popular culture narratives are a type of gamification that can help create a more immersive and memorable learning experience [5].

Simulation in medical education relies on the development of a fiction contract between the facilitators and the learners. Fiction contracts are more easily created when the simulation closely resembles a realistic experience [6]. Using popular media themes, however, may pull the learner further away from perceiving a realistic scenario, inhibiting actual knowledge acquisition. Further, learners who do not resonate with the theme may have a more difficult time establishing their suspension of disbelief, and thus have a more difficult time engaging with and learning from the simulation.

The purpose of this pilot was to explore the effects of using a Star Wars theme in an emergency medicine simulation with regard to two primary outcomes: (1) the subjective learner experience related to enjoyment, engagement, and perceived learning; and (2) knowledge acquisition as evidenced by pre- and post-test scores. The authors hypothesize that learners with a preexisting affinity for Star Wars will have greater subjective experience as well as better knowledge acquisition than learners who do not have such a preexisting preference.

## Materials and methods

This study took place during an emergency medicine didactic conference day at a community-based residency program. All simulations used the Star Wars mythos as a framing tool for the scenarios, and residents and faculty were encouraged to dress in costumes or simply related clothing for the event. Simulation scenarios were created through the usual process for simulation days at our institution by a simulation fellowship-trained, American Board of Emergency Physicians-certified emergency physician.

After arrival, residents were invited to participate in the study, and those who opted in filled out the pre-test. Residents were split into three teams to compete in a gamified simulation focused on sports medicine topics with a Star Wars theme. The day consisted of three simulation cases and a procedural obstacle course. The simulation cases included a pediatric patient with a splenic rupture leading to cardiac arrest, an expandable neck hematoma with an unstable pelvic fracture, and a tibial plateau fracture with compartment syndrome requiring fasciotomy. The back story of each case was centered around the Star Wars theme and was scored based on both medical and communication-based critical actions. During the procedural obstacle course, residents progressed through stations using Star Wars-themed rhyming clues that directed them to the next location. Each clue referenced characters or concepts from the Star Wars universe, such as a fracture being caused by a light saber battle, and required participants to find the correct activity/procedure to solve or perform. The components of the obstacle course included auricular hematoma drainage, identifying a ruptured Achilles tendon on ultrasound, applying the appropriate splint, lateral canthotomy, dental fracture matching with the appropriate fracture name and management, and X-ray identification, where residents had to name the fracture as well as the correct type of splint. This station was scored based on the timing of when all of the stations were completed. The critical actions from the simulation cases and the timing from the procedural obstacle course were combined to determine the winning team for the event. Both the simulation stations and the procedural obstacle course had time built in for debriefing. After the event, the participants completed the medical knowledge-based post-survey, which covered topics from the day.

Data analysis

Data was collected via an anonymous electronic survey pre- and post-simulation (Appendices A-B). The survey contained three sections. First was the collection of demographics, their self-generated anonymous ID for survey linking, and their pre-existing opinion of Star Wars ("I like Star Wars"/"I am neutral about Star Wars"/“I dislike Star Wars”). The second section surveyed their overall experience regarding the theme and use of gamification. In this part, participants rated their agreement on a 5-point Likert scale (Disagree, Slightly Disagree, Neutral, Slightly Agree, Agree) with statements assessing the enjoyment, engagement, and ease of material learning of this learning experience. The third section was the knowledge assessment - a 10-question multiple-choice test on the medical topics covered in the simulation. Participants completed this test immediately before (pre-test) and after (post-test) the simulation event. Scores were calculated as a percentage of correct answers.

Participants were stratified into two cohorts based on their Star Wars opinion: "Pro-Star Wars" (those who reported "I like Star Wars") and "Neutral to Star Wars" (those who reported "I am neutral about Star Wars"). No respondents selected the “I do not like Star Wars” response. Descriptive statistics (means and standard deviations) were calculated for pre- and post-test scores. A two-tailed paired t-test was used to analyze the within-cohort improvement from pre- to post-test. An unpaired t-test was used to compare the magnitude of improvement between the two cohorts. A p-value of <0.05 was considered statistically significant. Confidence intervals (95% CIs) were calculated for score improvements.

## Results

Participant characteristics

Eleven participants completed both the pre-test, post-test, and survey. The cohort was divided into "Pro-Star Wars" (n=7) and "Neutral to Star Wars" (n=4). One additional participant (ID 13) was excluded from the primary analysis due to incomplete data.

Knowledge acquisition

Both cohorts demonstrated an improvement in test scores from pre-test to post-test (Table 1). The "Pro-Star Wars" cohort improved from a mean of 81.0% to 90.0%, an average improvement of 8.57 percentage points (95% CI: -1.32, 18.46; p=0.078). The "Neutral to Star Wars" cohort improved from a mean of 80.0% to 87.5%, an average improvement of 7.50 percentage points (95% CI: -27.79, 45.78; p=0.54). The difference in the magnitude of improvement between the two cohorts was 1.07 percentage points, which was not statistically significant (95% CI: -16.36, 18.51; p=0.8925).

**Table 1:** Survey results for knowledge change between pre- and post-test, as well as subjective experience from the post-test. Responses of “agree” and “slightly agree” were considered positive affirmations.

	Pro-Star Wars	Neutral to Star Wars
Number of participants	7	4
Pre-test average	81.%	80.0%
Post-test average	90.0%	87.5%
Survey Item: Theme Implementation made…		
…the event more enjoyable	100%	75%
…the event more engaging	100%	50%
…learning the material easier	85%	0%
Survey Item: General Gamification made…		
…the event more enjoyable	100%	100%
…the event more engaging	100%	75%
…learning the material easier	85%	75%

Subjective thematic experience

The survey results revealed an interesting distinction in the subjective experience between the two groups (Table 1). Affirmative responses included “Agree” and “Slightly Agree.” All participants in the "Pro-Star Wars" cohort agreed that the Star Wars theme made the learning experience more engaging and enjoyable. In contrast, the "Neutral to Star Wars" cohort gave more mixed answers, including “Neutral,” "Slightly Disagree," and "Disagree" on these items, particularly for the statement "Learning the material easier." In terms of gamification in general (separate from the Star Wars theme), both cohorts had more uniformly positive responses.

## Discussion

This study offers early evidence that a Star Wars-themed simulation can support medical education. The main finding is that both groups of learners, regardless of their initial feelings about the theme, showed a minor but statistically insignificant improvement in test scores after the intervention. This implies that the main educational value of the simulation remained unaffected by the theme implementation, even for those who were neutral to the theme.

The secondary finding focuses on learner engagement. As expected, participants who enjoyed Star Wars found the themed event more fun and engaging. This likely translates into the group also feeling like they learned the material more easily through the theming. However, the "Neutral to Star Wars" group found both the theme implementation and the use of gamification to be overall less enjoyable and engaging. Especially regarding the Star Wars theme, the implementation of that did not help make learning material easier. It is unable to be seen if this experiential difference led to a difference in actual knowledge acquisition, however.

It can be seen that students and learners appreciate the connection from classroom education with clips from popular media [7], but these are often brief anecdotes and not present for the duration of the educational activity. In our study, the Star Wars theme was ever-present, likely leading to stronger feelings regarding the theming and its effect on engagement and enjoyment. Other medical shows have been successfully used to bridge the gap from classroom education to real-world scenarios [8-10]. However, popular media here is being used to facilitate the transfer of knowledge to the clinical realm; hence, the focus on popular media is already set in a medical environment. Our study is attempting to use popular media as a framing mechanism in a simulated medical situation to draw the learner in, improve the fiction contract, and direct focus towards the educational content.

There is very limited evidence of using a non-medical popular media theming for simulation in medical education. The results here show a trend towards increased engagement, enjoyment, and perceived easier learning if the individual likes the media theming being used. However, there is an opposing trend for those who are neutral to the theme, feeling as if the theme adds little to the simulation education experience. It has been shown that increased engagement from learners in simulation does lead to increased knowledge acquisition [11,12]. Interestingly, in our results, however, there was little difference in the groups' pre- and post-test knowledge assessments, though this was not statistically significant. The sample sizes of this pilot study, however, prohibit drawing a true connection between theming and educational outcomes.

These results imply real-world implications for instructional design. They suggest that educators may not need to shy away from specific themes that might divide opinions, fearing that it will hurt some students’ learning. While tailoring the theme to the group that is to be taught is the most likely to improve subjective engagement and enjoyment, it is also possible that a well-crafted gamified activity can aid learning even if the theme is not universally liked. However, to fully support this theory, further research regarding popular media integration into medical education simulations needs to be done.

This study has several limitations, most notably of which is the small sample size, reducing statistical power and making generalizability difficult, as represented by the wide CIs. Another limitation is the absence of a control group that participated in a similar, non-themed simulation. Finally, the pre-/post-test design was a measure of short-term knowledge improvement, and future studies could benefit from long-term knowledge retention. There is a paucity of literature on the specific topic of popular media theming in simulation-based medical education. While the results here are too underpowered to be statistically significant, they imply an underlying trend towards the popular media theme itself having an effect on the education of the individual learners that would be beneficial to explore in future, larger studies.

## Conclusions

In this pilot study, a Star Wars-themed simulation was implemented as an interactive, educational activity for emergency medicine residents. While existing opinions about the theme affected enjoyment, they did not have a major impact on the learning gains. This supports the idea of using themed gamification in medical education as a way to boost engagement among learners who already identify as liking the theme prior to the educational activity. Future studies should include larger, randomized controlled trials with a variety of themes to better understand the connection between narrative, engagement, and learning.

## Figures and Tables

**Figure 1:**
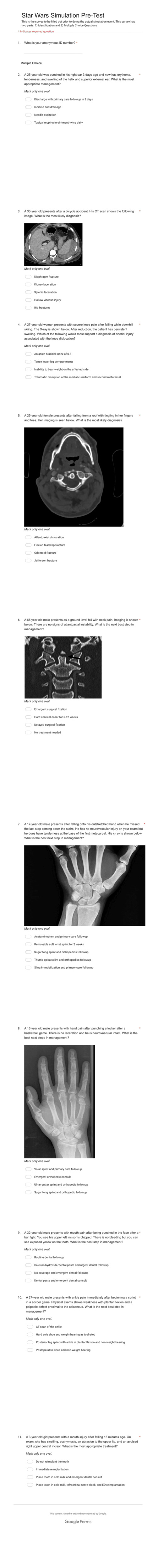
Pre-test survey given prior to intervention consisting of demographic information collection and baseline knowledge assessment.

**Figure 2:**
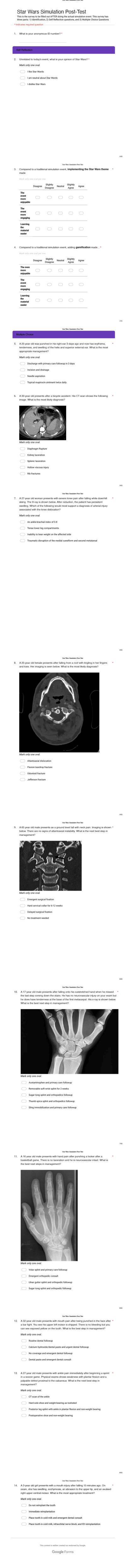
Post-test given after intervention, including the demographic collection for anonymous response linking, subjective self-reflective responses, and the repeated knowledge test.

## Appendices

Appendix A 

Appendix B 

## References

[1] Elendu C, Amaechi DC, Okatta AU, Amaechi EC, Elendu TC, Ezeh CP, Elendu ID (2024). The impact of simulation-based training in medical education: a review. Medicine (Baltimore).

[2] Rutledge C, Walsh CM, Swinger N (2018). Gamification in action: theoretical and practical considerations for medical educators. Acad Med.

[3] Do M, Sanford K, Roseff S, Hovaguimian A, Besche H, Fischer K (2023). Gamified versus non-gamified online educational modules for teaching clinical laboratory medicine to first-year medical students at a large allopathic medical school in the United States. BMC Med Educ.

[4] Gue S, Ray J, Ganti L (2022). Gamification of graduate medical education in an emergency medicine residency program. Int J Emerg Med.

[5] Dalavaye N, Baskaran R, Mukhopadhyay S, Gamage MP, Ng V, Sharif H, Rutherford S (2023). Exploring the educational value of popular culture in web-based medical education: pre-post study on teaching jaundice using “The Simpsons”. JMIR Med Educ.

[6] Sharma H, Patil AD, Baviskar A (2023). Fiction contract: its importance in simulation-based medical education. Int J Basic Clin Pharmacol.

[7] Hoffman BL, Hoffman R, Wessel CB, Shensa A, Woods MS, Primack BA (2018). Use of fictional medical television in health sciences education: a systematic review. Adv Health Sci Educ Theory Pract.

[8] Jerrentrup A, Mueller T, Glowalla U, Herder M, Henrichs N, Neubauer A, Schaefer JR (2018). Teaching medicine with the help of "Dr. House". PLoS One.

[9] Hirt C, Wong K, Erichsen S, White JS (2013). Medical dramas on television: a brief guide for educators. Med Teach.

[10] Osborne F, Harrison M, Fisher J, Bateman B (2021). Using medical reality television as a technology-enhanced learning strategy to provide authentic patient care experiences during clinical placements: a case study research investigation. BMC Med Educ.

[11] Padgett J, Cristancho S, Lingard L, Cherry R, Haji F (2019). Engagement: what is it good for? The role of learner engagement in healthcare simulation contexts. Adv Health Sci Educ Theory Pract.

[12] Raleigh MF, Wilson GA, Moss DA (2018). Same content, different methods: comparing lecture, engaged classroom, and simulation. Fam Med.

